# Marital status and occupation in relation to short-term case fatality after a first coronary event - a population based cohort

**DOI:** 10.1186/1471-2458-10-235

**Published:** 2010-05-10

**Authors:** Sofia Gerward, Patrik Tydén, Gunnar Engström, Bo Hedblad

**Affiliations:** 1 Department of Clinical Sciences in Malmö, Cardiovascular Epidemiology, Skåne University Hospital, Malmö, Sweden; 2 Department of Cardiology, Skåne University Hospital, Malmö, Sweden

## Abstract

**Background:**

Although marital status and low occupation level has been associated with mortality, the relationship with case fatality rates (CFR) after a coronary event (CE) is unclear. This study explored whether incidence of CE and short-term CFR differ between groups defined in terms of marital status and occupation, and if this could be explained by biological and life-style risk factors.

**Methods:**

Population-based cohort study of 33,224 subjects (67% men), aged 27 to 61 years, without history of myocardial infarction, who were enrolled between 1974 and 1992. Incidence of CE, and CFR (death during the first day or within 28 days after CE, including out-of-hospital deaths) was examined over a mean follow-up of 21 years.

**Results:**

A total of 3,035 men (6.0 per 1000 person-years) and 507 women (2.4 per 1000) suffered a first CE during follow-up. CFR (during the 1^st ^day) was 29% in men and 23% in women. After risk factor adjustments, unmarried status in men, but not in women, was significantly associated with increased risk of suffering a CE [hazard ratios (HR) 1.10, 95% CI: 0.97-1.24; 1.42: 1.27-1.58 and 1.77: 1.31-2.40 for never married, divorced and widowed, respectively, compared to married]. Unmarried status, in both gender, was also related with an increased CFR (1^st ^day), taking potential confounders into account (odds ratio (OR) 2.14, 95% CI: 1.63-2.81; 1.91: 1.50-2.43 and 1.49: 0.77-2.89 for never married, divorced and widowed, respectively, compared to married men. Corresponding figures for women was 2.32: 0.93-5.81; 1.87: 1.04-3.36 and 2.74: 1.03-7.28. No differences in CFR (1^st ^day) were observed between occupational groups in neither gender.

**Conclusions:**

In this population-based Swedish cohort, short-term CFR was significantly related to unmarried status in men and women. This relationship was not explained by biological-, life-style factors or occupational level.

## Background

Incidence and mortality of acute coronary events (CE) have been associated with low occupation level and being unmarried [1-6]. However, it is still controversial whether short-term case-fatality rates (CFR), including out-of-hospital deaths, after suffering a first CE varies between these groups. Approximately 40-60% of all coronary deaths occurs out-of-hospital [7,8]. We have in previous studies from the city of Malmö, Sweden, reported marked differences in short-term CFR after myocardial infarction (MI) between residential areas defined by their socioeconomic circumstances [9,10]. Annual income has also been inversely related to incidence of MI and short-term CFR in this city [8]. Socioeconomic differences in short-term CFR were also reported in a study from northern Sweden [11]. A French population-based case-control study reported higher risk of out-of hospital sudden cardiac arrest in unmarried subjects [12]. The reasons for these relationships are still unclear, but might be related to higher prevalence of risk factors for CHD among lower socioeconomic groups [13].

We used data from a population-based cohort of 33,224 middle-aged subjects, without history of myocardial infarction, from the city of Malmö, Sweden, to prospectively examine the relationships between marital status and occupation level, respectively, and incidence of CE, taking several biological and life-style risk factors into account. We also explored the CFR during the 1^st ^day and within 28-days, including deaths occurring out-of-hospital.

## Methods

### Study population

The 'Malmö Preventive Project (MPP)' included a screening examination of the adult population in the city of Malmö, Sweden. Complete birth cohorts were invited to the screening, which was performed with the purpose of identifying high-risk individuals for cardiovascular diseases [14]. Between 1974 and 1984, 22,444 men, aged 27-61 years, were examined, and 10,902 women, 28-58 years of age, were examined between 1977 and 1992. Overall attendance rate was 71% (71.4% in men and 70.8% in women, range 64-78%, somewhat differing between years). Mean age was 49.7 ± 7.4 years in women and 43.7 ± 6.6 years in men. Information on non-participation in the MPP has been reported previously [15]. We excluded subjects with history of myocardial infarction before the baseline examination (i.e. 97 men and 25 women, respectively) according to regional or national myocardial infarction (MI) registers [16,17] leaving 33,224 subjects. Of these, information on marital status and occupation was available for 32,857 and 32,916 subjects respectively. The health service authority of Malmö approved and funded the screening program. Incidence of cardiovascular disease in this cohort was monitored by data linkage with national registers after due approval by the regional research ethical committee Malmö/Lund, Lund University, Lund, Sweden.

### Sociodemographic characteristics

Information about marital status and occupation was retrieved by data linkage with the Swedish national census investigations performed in 1970, 1980, 1985 and 1990 which are total registers of the Swedish population in those years (in Swedish: Folk och Bostadsräkning FoB, Statistics Sweden, http://www.scb.se). Classification was carried out in accordance to the census that was closest to the date for the health examination. Marital status was categorized into never married, married, divorced and widowed. Among subjects who suffered a first CE during the follow-up, information about marital status at the baseline examination was available for 3,435 (97%) subjects.

The categorization of occupational status was based on a classification system which takes into account the educational background needed to qualify for a particular job, any additional prerequisites, the level of responsibility the job entails and the specific tasks to be performed. Occupation was categorized into high-level nonmanual workers (i.e. nonmanual work with at least six years post high-school education), medium-level nonmanual workers (i.e. nonmanual work with three to five year post high-school education), low-level nonmanual workers (i.e. nonmanual work with two years or less education post high-school), skilled manual workers (i.e. manual work and at least two years post high-school education), unskilled manual workers (i.e. manual work with less than two years post high-school education) and self-employed (i.e. entrepreneurs and farmers). Information on occupation at baseline was available in 3,504 (99%) subjects who had a first CE during the follow-up period. Of those, we excluded 181 subjects classified as "retired" (i.e. early retired people with disability pension or social support pension due to long-term unemployment) and "other" (i.e. students and housewives without classification) [5].

### Baseline examinations

Blood pressure (mmHg) in supine position was measured twice, after 10-min rest, in the right arm. The average of two measurements was used. Information on smoking habits (categorized as smokers and non-smokers), physical inactivity during leisure-time (categorized as sedentary or not), history of angina pectoris, stress at work, alcohol consumption and current use of blood pressure lowering medication was assessed from a self-administered questionnaire [14]. Subjects who confirmed doctor's diagnosis of angina pectoris or who used nitrates were considered to have angina pectoris. Subjects with affirmative answers to the questions "Are you stressed at work?" and/or "Are you working much over-time?" were considered having stressful work. Problematic alcohol behaviour was assessed by means of the modified shortened version of the Michigan Alcoholism Screening Test. Subjects with > 2 (of 9) affirmative answers were considered to have high alcohol consumption [15]. Body mass index (BMI) was calculated as weight/height^2 ^(kg/m^2^).

Blood samples were taken after an overnight fast. Serum cholesterol, triglycerides and glucose was analyzed with standard methods at the laboratory of Malmö University Hospital. Prevalence of diabetes was defined as either a fasting blood glucose ≥ 6.1 mmol/L, a 2-hour glucose value ≥ 10.0 mmol/L (glucose load, 30 g/m^2 ^body surface area) during an oral glucose tolerance test or history or current treatment of the disease.

### Follow-up and Definition of End Points

Information on morbidity and mortality after the health examination was obtained by record linkage with the national MI register [15,16]. Death certificates used for the classification of the underlying cause of death and hospital diagnosis had been coded in accordance with the ninth and tenth version of the International Classification of Diseases, Injuries and Causes of Death (ICD). Information on emigration was retrieved by data linkage with the emigration register at the Swedish National Bureau of Statistics. All participants in the cohort were followed for mortality and incidence of CE until date of emigration or December 31, 2004. The numerator used for computation of incident CE are those who had acute MI (ICD-9 code 410 or ICD-10 code I2I) and those who; according to the death certificate, died of ischemic heart disease (ICD-9 codes 411-414 or ICD-10 codes I20, I24-25) before reaching hospital. Cause of deaths was based on autopsy in 73% of the cases (n = 1143). Cause of deaths in cases not autopsied was based on examinations in-hospital before death (n = 219), on findings from examinations outside hospital before death (n = 63), and on other sources (n = 28). CFR (during the 1^st ^day) include all cases that died on the day of the CE, in-hospital or outside hospital. CFR (within 28-days) include out-of-hospital deaths and deaths within 28 days after hospitalization.

### Statistics

For continuous baseline variables, a General Linear Model (GLM) was used to calculate age-adjusted values for the categories of marital status and occupation. For categorical baseline variables, an age-adjusted logistic regression analysis was used. We calculated CE free survival in groups of marital status with the Kaplan-Meier method. Four categories of marital status were used with married as the reference group, and six categories of occupation level using high-level nonmanual occupation as the reference group. This method was also used to illustrate the 28-day survival after CE. Sex-specific Cox proportional hazards models were used to estimate the hazards ratios (HR) of incident CE, with adjustments for cardiovascular risk factors.

Sex-specific logistic regression analysis was used to analyze the relationship between marital status and occupation level, and CFR during the 1^st ^day and within 28-days, respectively. First, the models were adjusted for age at first CE. Then, in a second model additional adjustments were made for date of first CE, biological risk factors (i.e. systolic blood pressure, blood pressure lowering medication, diabetes, total cholesterol, log transformed triglycerides, BMI, smoking, angina pectoris,) and life-style factors (physical inactivity, stressful work, problematic alcohol behaviour). Finally, marital status and occupation, respectively, was entered into the model. BMI, systolic blood pressure, cholesterol, log transformed triglycerides, age, date of first CE were entered into the models as continuous variables. Smoking, blood pressure-lowering medication, diabetes, angina pectoris, physical inactivity, stressful work, problematic alcohol behaviour, occupation, marital status were entered as categorical variables. A *p*-value ≤ 0.05 is considered to be statistically significant. SPSS version 15.0 was used for all statistical analyses.

## Results

During a mean follow-up of 22.5 ± 6.2 years in 22,347 men, 3,035 (13.6%) subjects had an incident CE. In women, 507 (4.7%) CE was recorded in 10,877 subjects during 19.2 ± 5.3 years of follow-up. Incidence of CE was 6.05 and 2.43 per 1000 person-years, respectively, among men and women. Among men 872 (28.7%) died the first day and another 1,007 (33.2%) within 28 days. Corresponding figures for women were 116 (22.9%) and 136 (26.8%), respectively.

### Baseline characteristics in relation to marital status and occupation

The baseline characteristics in subjects who subsequently had a CE are presented in Additional file 1, in relation to marital status. Never married were significantly younger than married men. Several cardiovascular risk factors showed significant differences between the categories of marital status, and the risk factor profiles were generally less favourable in men from the unmarried groups.

Baseline characteristics in relation to occupational level in subjects who subsequently had a CE are shown in Additional file 2. In summary, prevalence of smoking and stress at work in men were significantly higher in skilled- and unskilled workers compared to the reference group (i.e. high-level nonmanual).

### Incidence of CE and short term prognosis in relation to marital status and occupation

#### Marital status

**Figure 1:**
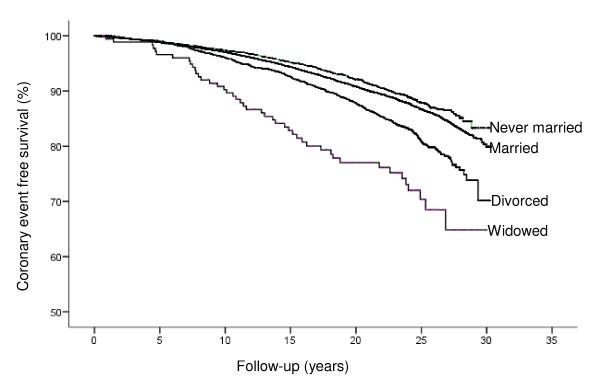
**Crude coronary event free survival in relation to marital status in men**.

Crude CE free survival in relation to marital status in men is shown in Figure 1. The adjusted risk was significantly increased in never married, divorced and widowed men compared to married men, which remained after taking potential biological- and life style factors into account (HR, 1.21; 95% confidence interval (CI):1.08-1.35; 1.46:1.31-1.62 and 1.74:1.29-2.34, respectively, for never married, divorced and widowed men), see Additional file 3. Further adjustment for occupational level only marginally changed the relationships. No similar association was observed for women.

**Figure 2:**
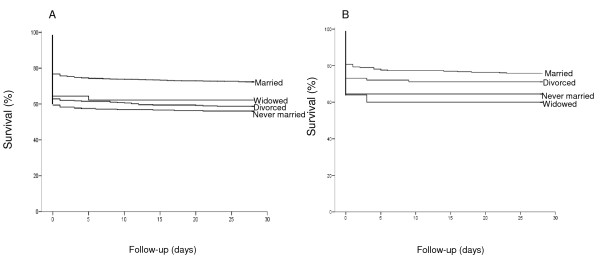
**Kaplan-Meier curves of 28-day survival following first coronary event in relation to marital status**. Men are presented in Figure 2A, and women in 2B.

In men, CFR (during the 1st day) was higher in never married or divorced cases. The increased odds ratios (OR) in these groups (i.e. 2.14; 95% CI: 1.63-2.81 and 1.91:1.50-2.43, respectively) were essentially unchanged after adjustments for several cardiovascular risk factors, including occupation (Table 1). Widowed men had also an increased risk, however not statistically significant after adjustment for age at incident CE (OR, 1.80; CI: 0.96-3.37). The relationship between marital status and CFR within 28 days in men (Figure 2A) was similar to that observed for CFR during the 1st day.

**Table 1:** CFR (the 1^st ^day) after first incident coronary event in relation to marital status

	Married		Never married		Divorced		Widowed	
Sex	(%)	**OR Ref**.	(%)	OR (95% CI)	(%)	OR (95% CI)	(%)	OR (95% CI)
No. first coronary events men	2091		362		434		45	
No. deaths men	486 (23.2)		147 (40.6)		161 (37.1)		16 (35.6)	
Men^a^		1		2.72 (2.14-3.46)		2.15 (1.72-2.69)		1.80 (0.96-3.37)
Men^a, b^		1		2.56 (1.99-3.29)		2.01 (1.60-2.54)		1.47 (0.78-2.79)
Men^a, b, c^		1		2.14 (1.63-2.81)		1.91 (1.50-2.43)		1.49 (0.77-2.89)
No. first coronary events women	343		31		104		25	
No. deaths women	66 (19.2)		11 (35.5)		28 (26.9)		9 (36.0)	
Women^a^		1		2.31(1.06-5.06)		1.55 (0.93-2.58)		2.36 (1.00-5.58)
Women^a, b^		1		2.21 (0.98-4.98)		1.78 (1.04-3.07)		2.43 (0.98-6.01)
Women^a, b, c^		1		2.32 (0.93-5.81)		1.87 (1.04-3.36)		2.74 (1.03-7.28)

Abbreviations: OR, Odds Ratio; CI, confidence interval.

OR calculated after adjustment for age at first coronary event^a ^and for date of first coronary event, systolic blood pressure, blood pressure medication, diabetes, cholesterol, log triglycerides, body mass index, angina pectoris, smoking, physical inactivity, stressful work, problematic alcohol behaviour^b ^and occupation^c^. The final model was based on 2772 men (738 deaths) and 437 women (96 deaths) with complete information on all covariates.

Among women, CFR (during the 1^st ^day) was higher in never married, divorced and widowed compared to married cases. After correction for biological-, life style factors and occupation the increased risk remained in those who were divorced or widowers (OR, 1.87, 95% CI:1.04-3.36 and 2.74: 1.03-7.28, respectively, Table 1). The relationship between marital status in women and CFR (within 28-days) did not reach significance after adjustments for risk factors, (Figure 2B).

#### Occupational level

**Figure 3:**
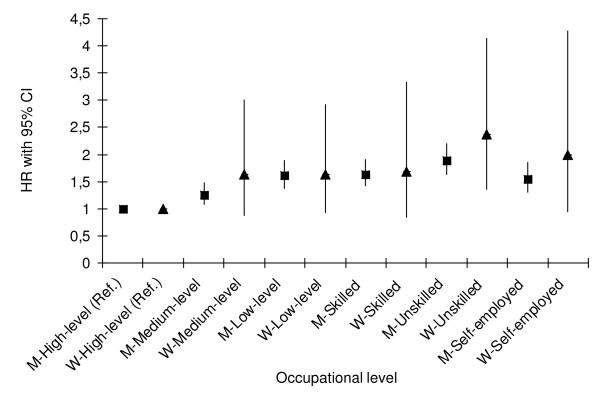
**Hazard ratios for incident coronary event by occupational level in men (M) and women (W)**. Age-adjusted hazard ratios (HR), with 95% confidence interval (CI) is presented, using high-level non-manual occupation level as the reference group.

As shown in Figure 3, the age-adjusted HR for incident CE was significantly higher in men belonging to all other occupational levels compared to men with high-level nonmanual occupation. Further adjustments for biological- and life-style factors and marital status did not substantially change this relationship (data not shown). A similar association between occupational level and incident CE was observed among women (Figure 3), however, this was not significant after correction for biological- and life-style factors (data not shown).

CFR (during the 1^st ^day and within 28-days) was not related to occupational level in men or women. Compared to high-level nonmanual workers, the highest age-adjusted OR for CFR (during the 1^st ^day) was observed among unskilled manual male workers (OR, 1.25: 0.90-1.73). Similar findings were observed among female unskilled manual workers (data not shown).

## Discussion

After an average of 21 years of follow-up, 14% of the men, and 5% of the women experienced an incident CE. In accordance with previous studies [2,3,5], incidence of CE among men was substantially higher in unmarried men and in men with low occupational level. Of those who suffered a CE, about 30% died within 28 days. Both in men and women, this proportion was significantly higher in unmarried compared to married cases. This finding is in line with previous studies from US [12] and Greece [18].

### Mechanisms

The increased incidence of CE and increased CFR in unmarried men was independent of many biological and life-style risk factors. However, there are several possible explanations for the relation between unmarried status and increased short-term case fatality rate. Patient delay in seeking care has been found to be associated with living alone, which may influence the proportion being treated with thrombolysis and invasive cardiac procedures [19]. There is also a possibility for higher prevalence of other diseases among unmarried subjects/patients which might affect survival. Social support is intimately linked to marital status and the social support offered by marriage seems to exert a protective effect at least for men, in reducing incidence and case fatality rates after CHD [20,21]. There are also studies implying that low-grade systemic inflammation could contribute to the increased cardiovascular risk in manual workers and divorced men [2].

Whether there are differences with regard to pre-hospital, in-hospital and post-CE treatment between marital status groups is not yet thoroughly investigated. The organization of primary care is similar across different residential areas in Malmö. By using recommended clinical guidelines one would expect similar principles for follow-up and treatment for married and unmarried men. Among patients dying pre-hospital from MI in Malmö, 72% in the low-income groups compared to 59% in the high-income group, had been in contact with the medical services within the last three months [8]. Whether these differences also exist between marital status groups has not been studied to our best knowledge. Studies have shown that a successful reduction of high cholesterol levels was associated with younger age and longer education in a primary health care-based programme for cardiovascular prevention [22]. Even though married subjects had higher cholesterol levels in that study, marital status was not associated with lipid reductions at follow-up. It has also been shown that smoking cessation rates are lower in single women and women from lower socioeconomic position (SEP) and psycho-social circumstances [23]. Whether unmarried subjects more often have larger infarctions or higher prevalence of left ventricular hypertrophy, known risk factors for cardiac death, remains to be evaluated. Even though there are indications that inferior SEP more often have transmural infarctions, higher incidence of heart failure [24,25] and lower access to invasive cardiac procedures [26,27], it is unclear whether this is true for unmarried subjects. Since most of our cases died out-of-hospital, it is impossible to collect information about these circumstances.

We found no significant relationship between unmarried status and incident CE in women. Assuming an age-adjusted HR of 1.2 (i.e. the point estimate observed in the present study) when comparing divorced and married women the statistical power in our study was only 50%. Corresponding power for men was as high as 92%. Hence, it is likely that the absence of a significant relation in women was explained by limited statistical power.

### Comparison with other studies

A recent case-control study of enrolees in a health maintenance organization, reported a higher proportion of unmarried individuals among cases with sudden cardiac arrest [12]. In that study, cases with previous MI or signs of heart disease were included and the study population comprised about 60% of patients with existing heart disease [12]. Similarly, in a Greek study including hospitalized patients due to an acute coronary syndrome, never married had 2-3 times higher risk of dying in-hospital or within 30-days. However, in that study patients who died out-of-hospital and patients dying the first three days in-hospital were not included and no gender-specific analysis was carried out [18]. In an Australian WHO MONICA study from the 1980s, unmarried men had significantly higher 28-day CFR than their married counterparts [28]. Most of the observed differences in that study were concentrated in the first 24 hours after onset of MI; however, cardiovascular risk factors were not taken into account.

A study from northern Sweden, including all first MI events aged 25 to 64 years, showed lower 28-day case fatality rate among manual compared to non-manual workers [11]. A previous study based on the same cohort, showed that male skilled and unskilled manual workers had higher cardiovascular mortality compared to high-level non-manual workers [5]. Absence of a corresponding relationship between occupation and short-term case fatality rate in the present study might implicate that occupational level is less important than marital status in terms of short-term CFR after a first CE.

### Limitations

Some methodological issues of our study need to be mentioned. This study includes cases who according to the national MI register been registered the first time as with a non-fatal MI or who have died due to IHD. This register covers the southern part of Sweden during the entire follow-up period. A validation study from the Swedish Hospital Discharge Register showed that the diagnosis "MI" was false in only 5% of the cases [16]. It has been estimated that 25% of patients having a MI are so called "silent cases" [29] and these patients are not included by definition. Another source of bias could possibly be that we included subjects who died out-of-hospital. However, in the present study almost 75% of all CHD deaths were based on autopsy. In addition, in the city of Malmö there is only one hospital for treating patients with acute CE which is strength of this study.

Information on risk factors was only collected at baseline and we do not have information on changes in biological and life-style risk factors during the follow-up period. About 25% of the screened subjects participated in various interventions to prevent cardiovascular disease and alcohol abuse [15]. However, population level comparisons with non-invited groups from the city of Malmö have not shown any reduced mortality from this prevention strategy [15].

Assessment of occupation level and marital status at baseline, instead of repeated measurements during follow-up, however, has been shown to be sufficient in a long-term follow-up study from this cohort [5].

Non-participants in MPP have generally been characterized by more adverse social factors which could perhaps underestimate our current findings [15]. This could also possibly explain the lack of association between occupation and CFR in the present study.

## Conclusions

In this population-based Swedish cohort, short-term CFR was related to marital status in men and women. Our findings suggest that being unmarried increases the risk of both having a first CE and dying from the event. As this relationship was independent of many biological- and life-style risk factors it may reflect a protective effect of marriage.

## Competing interests

The authors declare that they have no competing interests.

## Authors' contributions

SG participated in the design of the study, collected material, performed the statistical analyses and drafted the manuscript. GE and BH designed the study and drafted the manuscript and revised it critically. PT revised it critically. All authors read and approved the final manuscript.

## Pre-publication history

The pre-publication history for this paper can be accessed here:

http://www.biomedcentral.com/1471-2458/10/235/prepub

## Supplementary Material

Additional file 1 **Risk factors at baseline by marital status among men and women who had a first coronary event**. Table showing prevalence of cardiovascular risk factors (i.e. systolic blood pressure, blood pressure medication, diabetes, cholesterol, triglycerides, body mass index, history of angina pectoris, smoking, physical inactivity, stressful work, problematic alcohol behaviour) at the baseline examination by marital status in men and women. For statistical analysis married men and women, respectively, are used as the reference group, all data are adjusted for age at screening. Click here for file

Additional file 2 **Risk factors at baseline by occupational level among men and women who had a first coronary event**. Table showing prevalence of cardiovascular risk factors (i.e. systolic blood pressure, blood pressure medication, diabetes, cholesterol, triglycerides, body mass index, history of angina pectoris, smoking, physical inactivity, stressful work, problematic alcohol behaviour) at the baseline examination by occupational level in men and women. For statistical analysis high-level nonmanual group in men and women, respectively, are used as the reference group, all data are adjusted for age at screening. Click here for file

Additional file 3 **Incidence of first coronary events in relation to marital status at baseline in men and women**. Table showing hazard ratio (HR) for incident first CE in relation to marital status at baseline in men and women. HRs are presented as initially adjusted for age at screening^a^; secondly for additional risk factors adjustment^b^; and finally also for occupation level^c^. Click here for file

## References

[B1] Ebrahim S Wannamethee G McCallum A Walker M Shaper AG Marital status, change in marital status, and mortality in middle-aged British men Am J Epidemiol 1995 142 8 834 842 7572960 10.1093/oxfordjournals.aje.a117723

[B2] Engstrom G Hedblad B Rosvall M Janzon L Lindgarde F Occupation, marital status, and low-grade inflammation: mutual confounding or independent cardiovascular risk factors? Arterioscler Thromb Vasc Biol 2006 26 3 643 648 10.1161/01.ATV.0000200100.14612.bb 16357315

[B3] Hedblad B Jonsson S Nilsson P Engstrom G Berglund G Janzon L Obesity and myocardial infarction--vulnerability related to occupational level and marital status. A 23-year follow-up of an urban male Swedish population J Intern Med 2002 252 6 542 550 10.1046/j.1365-2796.2002.01069.x 12472916

[B4] Mackenbach JP Bos V Andersen O Cardano M Costa G Harding S Reid A Hemstrom O Valkonen T Kunst AE Widening socioeconomic inequalities in mortality in six Western European countries International journal of epidemiology 2003 32 5 830 837 10.1093/ije/dyg209 14559760

[B5] Nilsson PM Nilsson JA Ostergren PO Berglund G Social mobility, marital status, and mortality risk in an adult life course perspective: the Malmo Preventive Project Scand J Public Health 2005 33 6 412 423 10.1080/14034940510005905 16332606

[B6] Rosengren A Wedel H Wilhelmsen L Marital status and mortality in middle-aged Swedish men Am J Epidemiol 1989 129 1 54 64 2910072 10.1093/oxfordjournals.aje.a115124

[B7] Chambless L Keil U Dobson A Mahonen M Kuulasmaa K Rajakangas AM Lowel H Tunstall-Pedoe H Population versus clinical view of case fatality from acute coronary heart disease: results from the WHO MONICA Project 1985-1990. Multinational MONItoring of Trends and Determinants in CArdiovascular Disease Circulation 1997 96 11 3849 3859 9403607 10.1161/01.cir.96.11.3849

[B8] Rosvall M Gerward S Engstrom G Hedblad B Income and short-term case fatality after myocardial infarction in the whole middle-aged population of Malmo, Sweden Eur J Public Health 2008 18 5 533 538 18621776 10.1093/eurpub/ckn059

[B9] Gerward S Tyden P Hansen O Engstrom G Janzon L Hedblad B Survival rate 28 days after hospital admission with first myocardial infarction. Inverse relationship with socio-economic circumstances J Intern Med 2006 259 2 164 172 10.1111/j.1365-2796.2005.01594.x 16420545

[B10] Tyden P Engstrom G Hansen O Hedblad B Janzon L Geographical pattern of female deaths from myocardial infarction in an urban population: fatal outcome out-of-hospital related to socio-economic deprivation J Intern Med 2001 250 3 201 207 10.1046/j.1365-2796.2001.00877.x 11555123

[B11] Peltonen M Rosen M Lundberg V Asplund K Social patterning of myocardial infarction and stroke in Sweden: incidence and survival Am J Epidemiol 2000 151 3 283 292 10670553 10.1093/oxfordjournals.aje.a010204

[B12] Empana JP Jouven X Lemaitre R Sotoodehnia N Rea T Raghunathan T Simon G Siscovick D Marital status and risk of out-of-hospital sudden cardiac arrest in the population Eur J Cardiovasc Prev Rehabil 2008 15 5 577 582 10.1097/HJR.0b013e3283083e04 18756177

[B13] Kaplan GA Keil JE Socioeconomic factors and cardiovascular disease: a review of the literature Circulation 1993 88 4 Pt 1 1973 1998 8403348 10.1161/01.cir.88.4.1973

[B14] Berglund G Eriksson KF Israelsson B Kjellstrom T Lindgarde F Mattiasson I Nilsson JA Stavenow L Cardiovascular risk groups and mortality in an urban swedish male population: the Malmo Preventive Project J Intern Med 1996 239 6 489 497 10.1046/j.1365-2796.1996.483819000.x 8656142

[B15] Berglund G Nilsson P Eriksson KF Nilsson JA Hedblad B Kristenson H Lindgarde F Long-term outcome of the Malmo preventive project: mortality and cardiovascular morbidity J Intern Med 2000 247 1 19 29 10.1046/j.1365-2796.2000.00568.x 10672127

[B16] National Board of Health and Welfare, Center of Epidemiology Värdering av diagnoskvaliten för akut hjärtinfarkt i patientregistret 1987 och 1995 [The validity of the diagnosis acute myocardial infarction in the patient registry 1987 and 1995] Stockholm, Sweden 2000

[B17] Engstrom G Berglund G Goransson M Hansen O Hedblad B Merlo J Tyden P Janzon L Distribution and determinants of ischaemic heart disease in an urban population. A study from the myocardial infarction register in Malmo, Sweden J Intern Med 2000 247 5 588 596 10.1046/j.1365-2796.2000.00663.x 10809998

[B18] Panagiotakos DB Pitsavos C Kogias Y Mantas Y Zombolos S Antonoulas A Giannopoulos G Chrysohoou C Stefanadis C Marital status, depressive episodes, and short-term prognosis of patients with acute coronary syndrome: Greek study of acute coronary syndrome (GREECS) Neuropsychiatr Dis Treat 2008 4 2 425 432 18728739 10.2147/ndt.s2185 PMC2518381

[B19] Moser D Dnsc RN Kimble L PhD RN Alberts M Md F Alonzo A Croft J Dracup K Dnsc RN Reducing Delay in Seeking Treatment by Patients With Acute Coronary Syndrome and Stroke: A Scientific Statement From the American Heart Association Council on Cardiovascular Nursing and Stroke Council Circulation (New York, NY) 2006 114 2 168 182 10.1161/CIRCULATIONAHA.106.176040 16801458

[B20] Andre-Petersson L Hedblad B Janzon L Ostergren PO Social support and behavior in a stressful situation in relation to myocardial infarction and mortality: who is at risk? Results from prospective cohort study "Men born in " Malmo, Sweden Int J Behav Med 1914 13 4 340 347 10.1207/s15327558ijbm1304_9 17228992

[B21] Mookadam F Arthur HM Social support and its relationship to morbidity and mortality after acute myocardial infarction: systematic overview Arch Intern Med 2004 164 14 1514 1518 10.1001/archinte.164.14.1514 15277281

[B22] Hellenius ML Nilsson P Elofsson S Johansson J Krakau I Reduction of high cholesterol levels associated with younger age and longer education in a primary health care programme for cardiovascular prevention Scand J Prim Health Care 2005 23 2 75 81 10.1080/02813430510018365 16036545

[B23] Janzon E Engstrom G Lindstrom M Berglund G Hedblad B Janzon L Who are the "quitters"? a cross-sectional study of circumstances associated with women giving up smoking Scand J Public Health 2005 33 3 175 182 10.1080/14034940410019244 16040457

[B24] Barakat K Stevenson S Wilkinson P Suliman A Ranjadayalan K Timmis AD Socioeconomic differentials in recurrent ischaemia and mortality after acute myocardial infarction Heart 2001 85 4 390 394 10.1136/heart.85.4.390 11250961 PMC1729679

[B25] Tofler GH Muller JE Stone PH Davies G Davis VG Braunwald E Comparison of long-term outcome after acute myocardial infarction in patients never graduated from high school with that in more educated patients. Multicenter Investigation of the Limitation of Infarct Size (MILIS) Am J Cardiol 1993 71 12 1031 1035 10.1016/0002-9149(93)90568-W 8475864

[B26] Alter DA Naylor CD Austin P Tu JV Effects of socioeconomic status on access to invasive cardiac procedures and on mortality after acute myocardial infarction N Engl J Med 1999 341 18 1359 1367 10.1056/NEJM199910283411806 10536129

[B27] Rosvall M Chaix B Lynch J Lindstrom M Merlo J The association between socioeconomic position, use of revascularization procedures and five-year survival after recovery from acute myocardial infarction BMC Public Health 2008 8 44 10.1186/1471-2458-8-44 18241335 PMC2275258

[B28] Malcolm JA Dobson AJ Marriage is associated with a lower risk of ischaemic heart disease in men Med J Aust 1989 151 4 185 188 2629720 10.5694/j.1326-5377.1989.tb115986.x

[B29] Kannel WB Abbott RD Incidence and prognosis of unrecognized myocardial infarction. An update on the Framingham study N Engl J Med 1984 311 18 1144 1147 6482932 10.1056/NEJM198411013111802

