# Supplementary material for: Marital status and occupation in relation to short-term case fatality after a first coronary event - a population based cohort
Source: BMC Public Health. 2010 May 10;10:235. doi: 10.1186/1471-2458-10-235 (PMC2874781; doi:10.1186/1471-2458-10-235)
Supplement: Additional file 1 — Risk factors at baseline by marital status among men and women who had a first coronary event. Table showing prevalence of cardiovascular risk factors (i.e. systolic blood pressure, blood pressure medication, diabetes, cholesterol, triglycerides, body mass index, history of angina pectoris, smoking, physical inactivity, stressful work, problematic alcohol behaviour) at the baseline examination by marital status in men and women. For statistical analysis married men and women, respectively, are used as the reference group, all data are adjusted for age at screening. [file 1471-2458-10-235-S1.DOC]

**Additional file 1.** Risk factors at baseline by marital status among men and women who had a first coronary event.

|  | Married | | Never married | | Divorced | | Widowed | |
| --- | --- | --- | --- | --- | --- | --- | --- | --- |
|  | Men | Women | Men | Women | Men | Women | Men | Women |
| No. | 2091 | 343 | 362 | 31 | 434 | 104 | 45 | 25 |
| Age at screening, years | 47.3 ± 6.1 | 52.0 ± 5.4 | 44.5 ± 6.5*** | 50.6 ± 6.9 | 46.0 ± 5.2*** | 52.3 ± 5.1 | 51.0 ± 7.5*** | 54.4 ± 2.7* |
| Age at first CE, years | 63.2 ± 8.5 | 65.7 ± 6.8 | 60.2 ± 8.5*** | 64.5 ± 7.5 | 61.8 ± 8.2** | 65.2 ± 6.7 | 64.0 ± 7.7 | 64.9 ± 6.3 |
| Time to first CE from screening, years | 15.9 ± 7.1 | 13.7 ± 6.6 | 15.7 ± 6.8* | 13.9 ± 5.6 | 15.8 ± 6.8 | 12.9 ± 6.4 | 13.0 ± 6.8* | 10.5 ± 6.2 |
| Systolic blood pressure, mmHg | 132 ± 17 | 133 ± 18 | 134 ± 18*** | 136 ± 19 | 132 ± 17 | 126 ± 15*** | 140 ± 21** | 132 ± 15 |
| Blood pressure medication, n (%) | 170 (8.1) | 63 (18.4) | 24 (6.4) | 6 (19.4) | 25 (5.8) | 13 (12.5) | 4 (8.9) | 5 (20.0) |
| Diabetes^a^, n (%) | 175 (8.4) | 48 (14.0) | 40 (11.0)* | 4 (12.9) | 39 (9.0) | 10 (9.6) | 5 (11.1) | 8 (32.0)* |
| Cholesterol, mmol/l (x38.6=mg/dl) | 6.0 ± 1.1 | 6.4 ± 1.2 | 6.1 ± 1.3 | 6.2 ± 1.3 | 6.0 ± 1.1 | 6.3 ± 1.4 | 6.1 ± 1.1 | 6.6 ± 1.5 |
| Triglycerides, mmol/l (x88.5=mg/dl) | 1.8 ± 1.1 | 1.4 ± 0.8 | 1.8 ± 1.3 | 1.3 ± 0.7 | 1.9 ± 1.2 | 1.4 ± 0.6 | 1.8 ± 1.0 | 1.5 ± 0.9 |
| Body Mass Index, kg/m^2^ | 25.4 ± 3.3 | 25.6 ± 4.6 | 26.0 ± 4.3*** | 26.1 ± 5.8 | 25.2 ± 3.5 | 25.4 ± 4.3 | 25.6 ± 3.7 | 25.7 ± 4.1 |
| History of angina pectoris^b^, n (%) | 74 (3.5) | 14 (4.1) | 9 (2.5) | 2 (6.5) | 13 (3.0) | 6 (5.8) | 2 (4.4) | 1 (4.0) |
| Smoking, n (%) | 1261 (60.3) | 179 (52.2) | 233 (64.41) | 21 (67.7) | 325 (74.9)*** | 71 (68.3)** | 35 (77.8)* | 19 (76.0)* |
| Physical inactivity, n (%) | 1112 (53.2) | 62 (18.1) | 231 (63.8)*** | 6 (19.4) | 266 (61.3)*** | 14 (13.5) | 27 (60.0) | 1 (4.0) |
| Stressful work^c^ (%) | 759 (36.3) | 29 (8.5) | 71 (19.6)*** | 2 (6.5) | 148 (34.1) | 8 (7.7) | 11 (24.4) | 0 |
| Problematic alcohol behaviour, n (%) | 423 (20.2) | 22 (6.4) | 109 (30.1)** | 4 (12.9) | 167 (38.5)*** | 16 (15.4)** | 14 (31.1)* | 0 |
|  |  |  |  |  |  |  |  |  |

Abbreviations: CE, coronary event. Data are presented as standard deviation or as proportions. ^a^ Fasting blood glucose ≥ 6.1 mmol/l and/or 2-hour glucose values ≥ 10.0 mmol/l and/or treatment;^b^ History of angina pectoris or nitroglycerin treatment; ^c^ Stressed at work and/or working over-time. *p≤0.05; **p≤0.01, ***p≤0.001, adjusted for age at screening using married men and women, respectively, as the reference group. Triglycerides are log transformed in the calculations. Time to first coronary event adjusted for age at screening.
