# Supplementary material for: Marital status and occupation in relation to short-term case fatality after a first coronary event - a population based cohort
Source: BMC Public Health. 2010 May 10;10:235. doi: 10.1186/1471-2458-10-235 (PMC2874781; doi:10.1186/1471-2458-10-235)
Supplement: Additional file 3 — Incidence of first coronary events in relation to marital status at baseline in men and women. Table showing hazard ratio (HR) for incident first CE in relation to marital status at baseline in men and women. HRs are presented as initially adjusted for age at screeninga; secondly for additional risk factors adjustmentb; and finally also for occupation levelc. [file 1471-2458-10-235-S3.DOC]

**Additional file 3**. Incidence of first coronary events in relation to marital status at baseline in men and women.

|  | **Married** | | **Never married** | | **Divorced** | | **Widowed** | |
| --- | --- | --- | --- | --- | --- | --- | --- | --- |
|  | (%) | HR Ref. | (%) | HR (95% CI) | (%) | HR (95% CI) | (%) | HR (95% CI) |
| Men |  |  |  |  |  |  |  |  |
| No. at screening | 15944 |  | 3279 |  | 2644 |  | 179 |  |
| No. first CE | 2091(13.1) |  | 362 (11.0) |  | 434 (16.4) |  | 45 (25.1) |  |
| Incidence 1000 p-yrs | 5.70 |  | 4.92 |  | 7.80 |  | 13.3 |  |
| Age-adjusted ^a^ |  | 1 |  | 1.22 (1.09-1.36) |  | 1.56 (1.41-1.74) |  | 1.94 (1.45-2.61) |
| +RF-adjusted ^a,b^ |  | 1 |  | 1.21 (1.08-1.35) |  | 1.46 (1.31-1.62) |  | 1.74 (1.29-2.34) |
| +Occupation^a,b,c^ |  | 1 |  | 1.10 (0.97-1.24) |  | 1.42 (1.27-1.58) |  | 1.77 (1.31-2.40) |
| Women |  |  |  |  |  |  |  |  |
| No. at screening | 7369 |  | 901 |  | 1981 |  | 560 |  |
| No. first CE | 343 (4.7) |  | 31 (3.4) |  | 104 (5.2) |  | 25 (4.5) |  |
| Incidence 1000 p-yrs | 2.39 |  | 1.69 |  | 2.88 |  | 2.50 |  |
| Age-adjusted ^a^ |  | 1 |  | 0.98 (0.68-1.42) |  | 1.23 (0.98-1.53) |  | 0.82 (0.55-1.23) |
| +RF-adjusted ^a,b^ |  | 1 |  | 0.97 (0.67-1.40) |  | 1.00 (0.79-1.25) |  | 0.70 (0.46-1.06) |
| +Occupation ^a,b,c^ |  | 1 |  | 0.95 (0.64-1.41) |  | 1.05 (0.83-1.34) |  | 0.77 (0.50-1.21) |
|  |  |  |  |  |  |  |  |  |

Abbreviations: HR, Hazard Ratio; CI, confidence interval; CE, coronary event; p-yrs, person years; RF, risk factor.

HRs are presented as initially adjusted for age at screening ^a^ , secondly for systolic blood pressure, blood pressure medication, cholesterol,

log triglycerides, diabetes, body mass index, smoking, history of angina pectoris, physical inactivity, stressful work and problematic alcohol behaviour ^b^,

and finally also for occupational level^c^ .

The final model was based on 21256 men (2772 CE) and 9924 women (437 CE) with complete information on all covariates.
